# Collaboration With the State Department of Health

**DOI:** 10.1093/geroni/igab046.223

**Published:** 2021-12-17

**Authors:** Kathleen Unroe

**Affiliations:** Indiana University/Probari, Indianapolis, Indiana, United States

## Abstract

COVID-19 disproportionately affected older adults, creating opportunities for experts in geriatrics and gerontology to support public policy. In Indiana, the Probari team, composed of a geriatrician and a team of nurses with geriatrics and palliative care expertise, supported the state government response to long-term care facilities during the pandemic. The team was involved in helping coordinate all staff testing (534 nursing homes) by the State Department of Health in June and in August, prior to the Federal mandated testing and the distribution of antigen machines. The Probari team also fielded surveys on behalf of the State regarding staff attitudes towards testing and willingness to be vaccinated, to inform state policy and resource efforts. In addition, Probari collaborated with the State Department of Health and the Indiana National Guard by training over 1600 service members to provide non-clinical support in nursing facilities, and monitoring and evaluating that 3 month deployment.

